# Supplementary material for: Fiber-Rich Barley Increases Butyric Acid-Producing Bacteria in the Human Gut Microbiota
Source: Metabolites. 2021 Aug 22;11(8):559. doi: 10.3390/metabo11080559 (PMC8399161; doi:10.3390/metabo11080559)
Supplement: Supplementary file 1 [file metabolites-11-00559-s001.zip › metabolites-1345781-supplementary.pdf]

metabolites

MDPI

Supplementary Material

Fiber-Rich Barley Increases Butyric Acid-Producing Bacteria in the Human Gut Microbiota

Shohei Akagawa [1], Yuko Akagawa [1], Yoko Nakai [1], Mitsuru Yamagishi [1], Sohsaku Yamanouchi [1], Takahisa Kimata [1], Kazushige Chino [2], Taiga Tamiya [3], Masaki Hashiyada [4], Atsushi Akane [4], Shoji Tsuji [1] and Kazunari Kaneko [1,*]

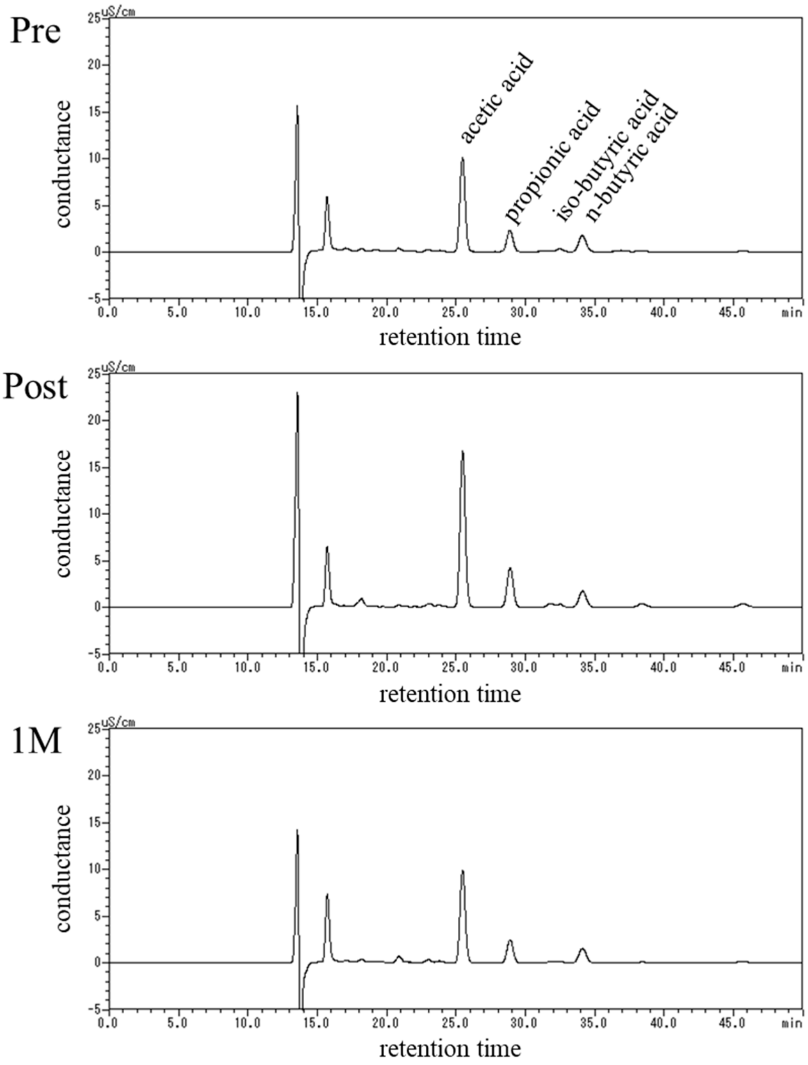

Figure S1. Representative chromatograms of short chain fatty acids at the three time points (pre, post, and 1 month) in one participant using high performance liquid chromatography.
